# Menstrual hygiene practices and associated factors among Indian adolescent girls: a meta-analysis

**DOI:** 10.1186/s12978-022-01453-3

**Published:** 2022-06-23

**Authors:** Jaseela Majeed, Prerna Sharma, Puneeta Ajmera, Koustuv Dalal

**Affiliations:** 1 School of Allied Health Sciences, Delhi Pharmaceutical Sciences and Research University, New Delhi, 110017 India; 2 Master of Public Health, Delhi Pharmaceutical Sciences and Research University, New Delhi, 110017 India; 3 Department of Public Health, School of Allied Health Sciences, Delhi Pharmaceutical Sciences and Research University, New Delhi, 110017 India; 4 Division of Public Health Science, Institute for Health Sciences, Mid Sweden University, Sundsvall, Sweden

**Keywords:** Menstrual hygiene management, Menstruation, Hygiene, Abnormalities, Disease, Morbidity, Intervention, Association

## Abstract

**Background:**

Menstrual hygiene management (MHM) and practices by adolescent females of low and middle-income countries (LMICs) are a severe public health issue. The current systematic review and meta-analysis aimed to estimate the pooled proportion of the hygiene practices, menstrual problems with their associated factors, and the effectiveness of educational interventions on menstrual hygiene among adolescent school girls in India.

**Methods:**

PRISMA checklist and PICO guidelines were used to screen the scientific literature from 2011 to 2021. The Newcastle–Ottawa Scale was used to assess the quality of studies. Four themes were developed for data analysis, including hygiene practices, type of absorbent used, menstruation associated morbidities and interventions performed regarding menstruation. Eighty-four relevant studies were included and a meta-analysis, including subgroup analysis, was performed.

**Results:**

Pooled data revealed a statistically significant increase in sanitary pad usage “(SMD = 48.83, 95% CI = 41.38–57.62, p < 0.00001)” and increased perineum practices during menstruation “(SMD = 55.77, 95% CI = 44.27–70.26, p < 0.00001)”. Results also reported that most prevalent disorders are dysmenorrhea “(SMD = 60.24, 95% CI = 50.41–70.06, p < 0.0001)”, Pre-menstrual symptoms “(SMD = 62.67, 95% CI = 46.83–78.50, p < 0.00001)”, Oligomenorrhea “(SMD = 23.57, CI = 18.05–29.10, p < 0.00001), Menorrhagia “(SMD = 25.67, CI = 3.86–47.47, p < 0.00001)”, PCOS “(SMD = 5.50, CI = 0.60–10.40, p < 0.00001)”, and Polymenorrhea “(SMD = 4.90, CI = 1.87–12.81, p < 0.0001)”. A statistically significant improvement in knowledge “(SMD = 2.06, 95% CI = 0.75–3.36, p < 0.00001)” and practice “(SMD = 1.26, 95% CI = 0.13–2.65, p < 0.00001)” on menstruation was observed. Infections of the reproductive system and their repercussions can be avoided with better awareness and safe menstruation practices.

**Conclusions:**

Learning about menstrual hygiene and health is essential for adolescent girls' health education to continue working and maintaining hygienic habits. Infections of the reproductive system and their repercussions can be avoided with better awareness and safe menstruation practices.

**Supplementary Information:**

The online version contains supplementary material available at 10.1186/s12978-022-01453-3.

## Plain language summary

Menstrual hygiene management (MHM) and practices by adolescent females of low and middle-income countries (LMICs) are severe problems for girls, parents, society, and policymakers. Menstrual-related problems are widespread among adolescent girls in India. Different menstrual abnormalities are found in different populations, suggesting socio-cultural and regional variation. Menstrual abnormalities and disorders are frequently linked to physical, mental, social, psychological, and reproductive issues, affecting adolescents' daily lives and their families lives by various psychosocial problems such as anxiety. We have the intention to compile, summarise, and critically analyse peer-reviewed and published scientific evidence from 2011 to 2021 on menstrual hygiene management methods used, most typical menstrual morbidities and their associated factors among Indian adolescent girls, and to evaluate the evidence for existing interventions like educational programs and absorbent distribution. Program planners and policymakers could use the findings of this study to build relevant initiatives to incorporate safe MHM in the country so that interventions can be designed taking into account the current needs of adolescent girls to reduce menstrual morbidities and improve their quality of life. A statistically significant improvement in knowledge and practice on menstruation was observed. Learning about menstrual hygiene and health is an essential aspect of adolescent girls’ health education to continue working and maintaining hygienic habits. Infections of the reproductive system and their repercussions can be avoided with better awareness and safe menstruation practices.

**Supplementary Information:**

The online version contains supplementary material available at 10.1186/s12978-022-01453-3.

## Introduction

The onset of menstruation (menarche) is one of the most significant transformations that girls go through during their adolescent years. Menstrual hygiene management (MHM) and practices by adolescent females of low and middle-income countries (LMICs) are a severe concern [1, 2]. Studies show that more than 50% of girls follow unsatisfactory MHM in LMICs, with rural areas having a higher percentage than urban areas [2–4]. Efficacious MHM requires access to clean absorbents and facilities for changing, cleaning or disposing of them as required, and soap and water for cleansing the body and the absorbents used during menstruation [5]. Hygiene-related practices during menstruation can lead to an increase in the risk of developing reproductive tract infections. Poor menstrual hygiene has a direct or indirect impact on the Sustainable Development Goals (3, 4, 5 and 6) and achieving them is critical for the overall development of these young adolescents and the country [6]. Although menstruation is a normal part of life, and it is associated with several myths and misunderstandings that might negatively affect health [7]. Menstruation is still seen as something repulsive or dirty in Indian society [8]. MHM is a severe problem in India for school-aged teenagers due to a lack of safe, sanitary facilities and limited or no sanitary hygiene products. As a result, many girls drop out of school due to a shortage of menstrual hygiene products and services [6].

Menstrual-related problems are widespread among adolescent girls in India. Different types of menstrual abnormalities are found in different populations, suggesting socio-cultural and regional variation [9]. Sixty-four per cent of girls have at least one menstrual-related issue [10]. In the age group of 10–19 years, poor menstrual hygiene and lack of self-care are critical drivers of morbidity and other problems. Some of the issues are urinary tract infections (UTI), scabies in the vaginal area, atypical abdominal pain, absence from school, and pregnancy complications [11]. Studies report that out of an estimated 113 million adolescent girls in India, around 68 million adolescent girls attend roughly 1.4 million schools. Poor MHM practices and cultural taboos are viewed to be barriers to their school attendance [12, 13]. Menstrual abnormalities and disorders are frequently linked to physical, mental, social, psychological, and reproductive issues, affecting adolescents’ daily lives and their families' live through various psychosocial problems such as anxiety [14].

To recognise the importance of promoting menstruation hygiene practices, the Government of India is undertaking many activities to raise awareness about the pivotal role that good MHM plays in enabling adolescent girls and women to achieve their full potential. A scheme was introduced in August 2011 to provide sanitary napkins at subsidised prices to adolescent girls in rural areas [15] as their reproductive health decisions today will impact the health and well-being of future generations and their community. On May 28, Menstrual Hygiene Day is observed to raise awareness of the problems that women and girls suffer as a result of their menstruation and to promote solutions that address these problems. Despite India’s efforts, a significant portion of adolescent girls lack prior knowledge of the menstrual cycle and associated hygienic habits, resulting in poor menstrual hygiene practices [16].

Numerous studies have been undertaken across India to assess the prevalence of MHM and its associated variables among adolescent schoolgirls. The results of these investigations were inconsistent and subject to significant variations. Also, various systematic reviews and meta-analyses have been conducted on MHM, incorporating either cross-sectional, case–control or interventional studies. To the best of our knowledge, we could not find any systematic review that has included all types of studies on MHM in the Indian context. Therefore, this systematic review and meta-analysis aimed to estimate the pooled proportion of the hygiene practices, menstrual problems with their associated factors, and the effectiveness of educational interventions on menstrual hygiene among adolescent schoolgirls in India. The intention is to compile, summarise, and critically analyse peer-reviewed and published evidence from 2011 to 2021 on MHM methods used, most typical menstrual morbidities and their associated factors among Indian adolescent girls, and evaluate the evidence for existing interventions like educational programs and absorbent distribution. Program planners and policymakers could use the findings of this study to build relevant initiatives to incorporate safe MHM in the country so that interventions can be designed taking into account the current needs of adolescent girls to reduce menstrual morbidities and improve their quality of life.

## Methods

The design and methodology for this systematic review and meta-analysis are developed and reported as per the “Preferred Reporting Items for Systematic Reviews and Meta-Analyses (PRISMA)” checklist [17]. The PICO guidelines [18] were used to determine eligibility requirements.

### Data sources and search strategy

One junior researcher (PS) and one senior researcher (PA) independently searched scientific literature in July 2021 to identify peer-reviewed published studies from 2011 to 2021 on menstrual hygiene, menstrual abnormalities and their associated factors and the effectiveness of education programmes among adolescent girls in India. Various combinations of keywords, “menstruation, hygiene, abnormalities, disease, morbidity, prevalence, associated factors, education, intervention and association” were administered. These search terms were combined with Boolean operators OR and AND to broaden or narrow the search. Additional studies were included by searching randomly in the databases. We limited the search results to Indian studies and further checked the original and review articles' reference lists that the initial search yielded to identify additional full-text articles. The search strategy used for different databases is presented in Table 1.

**Table 1:** Search strategy—PICO table and databases

Database	Framework	Search items	Number of articles
PUBMED	Population (P)	(Adolescent OR adolescence OR peer OR puberty OR school)	P: 7,770,604
	Intervention or condition (I)	AND (Menstruation OR menses)	I: 36,389 P + I: 12,788
	Control (C)	AND (Education OR programme OR management)	C: 5,674,219 P + I + C: 3,649
	Outcome (O)	AND (Hygiene OR sanitation OR hygienically OR Practice) AND (Infection OR abnormalities OR illness OR problem OR diseases OR morbidity) AND (Associated factors OR Modifiable factors OR Non modifiable factors)	O: 105,353 P + I + C + O: 114
	Timing (T)	(01/07/2011 to 01/07/2021)	P + I + C + O + T: 59
	Setting (S)	AND (India)	S: 646,516 P + I + C + O + S: 11
GOOGLE SCHOLAR	Population (P)	(Adolescent OR peer OR puberty OR school)	P: 56,60,000
	Intervention or condition (I)	AND (Menstruation OR menses)	I: 4,52,000 P + I: 2,05,000
	Control (C)	AND (Education OR programme OR management)	C: 74,50,000 P + I + C: 1,72,000
	Outcome (O)	AND (Hygiene OR sanitation OR Practice) AND (Infection OR abnormalities OR diseases OR morbidity) AND (Associated factors)	O: 30,90,000 P + I + C + O: 75,700
	Timing (T)	(01/07/2011 to 01/07/2021	T: 17,500
	Setting (S)	AND (India)	S: 54,90,000 P + I + C + O + T + S: 16,800
SCIENCE DIRECT	Population (P)	(Adolescent)	P: 417,843
	Intervention or condition (I)	AND (Menstruation OR menses)	I: 77,694 P + I: 12,901
	Control (C)	AND (Education OR management)	C: 1,000,000 + P + I + C: 8496
	Outcome (O)	AND (Hygiene OR morbidity) AND (Associated factors)	O: 627,667 P + I + C + O: 2863
	Timing (T)	AND (01/07/2011 to 01/07/2021	T: 1235
	Setting (S)	AND (India)	S: 1,000,000 + P + I + C + O + T + S: 238

### Eligibility criteria

*Inclusion criteria* Peer-reviewed journal articles written in English comprising original observational and interventional studies that reported menstrual morbidities such as dysmenorrhea, premenstrual syndrome (PMS), menorrhagia, polymenorrhagia, and oligomenorrhoea, along with articles incorporating the importance of education on menstruation from 2011 to 2021among Indian adolescent schoolgirls were included.

*Exclusion criteria* Systematic and narrative reviews, studies not performed on the Indian population, project reports, economic analysis, unpublished research and policy analysis have been excluded from this systematic review.

**Fig. 1:**
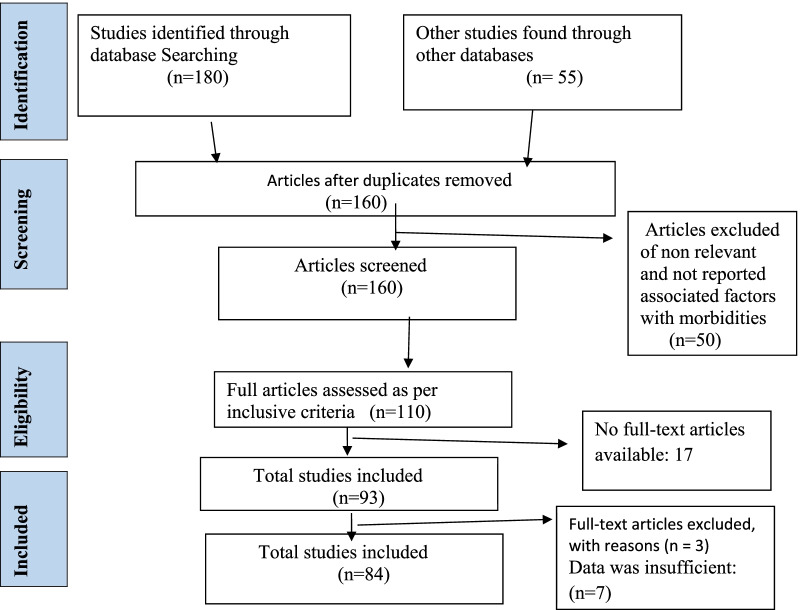
PRISMA flow chart

*Study selection* All the retrieved articles from each database found throughout the search process were noted, duplicates were deleted, and titles, abstracts, and complete publications were evaluated against eligibility criteria. The researchers screened the titles of the studies and their abstracts according to the inclusion and exclusion criteria. Two senior researchers rechecked data through repetitive meetings and all the disagreements and discrepancies were resolved by consensus. PRISMA flow diagram for data identification, screening, inclusion and exclusion is presented in Fig. 1.

### Data extraction

Data were extracted by designing the data extraction form, which includes the constituents like information about the publication, i.e., author(s) and year, location of study, the state of India where the study was performed, sample size, study procedure, menstrual hygiene practices, types of menstrual irregularities, common menstrual disorders and role of education on menstruation.

### Data synthesis

Revman 5.4 was used for statistical analysis (The Nordic Cochrane Centre, Copenhagen, Denmark). The meta-analysis included only papers that generated sufficient data on any pre-determined outcome measures. Because it is a more traditional methodology that compensates for the fact that study heterogeneity can differ more than by chance, a random-effects model was adopted to generate pooled effect sizes since we expected much heterogeneity. This approach assumes that the studies included are selected from ‘populations’ of research systematically different from one another (heterogeneity). The prevalence estimated from the included research varied due to random error among studies (fixed effects model) and genuine variance in prevalence from one study to the next. The data at the end of the intervention were retrieved for both the intervention and control groups. MS Excel was used for data synthesis.

### Quality assessment

The authors used the Newcastle–Ottawa Scale to assess the quality of studies included in the review [19]. The standard of observational and interventional studies was assessed using this scale. This scale uses a “star” system (with a maximum of nine stars for cross-sectional studies and seven stars for intervention-based studies) to rate the quality of a study in three areas: participant selection, study group comparability, and interest outcome determination.

## Results

### Characteristics and quality assessment of studies

The studies conducted from 2011 to 2021 intending to evaluate the menstrual hygiene practices, were screened and the most typical menstrual morbidities and their associated factors among young girls were identified. A total of 84 relevant studies reporting hygiene practices during menstruation by adolescent girls and menstrual morbidities and associated factors in India were obtained after removal of duplication and studies that did not fulfil inclusion criteria. Data collected from 84 studies were scrutinised and codes were created based on our objective and heterogeneity. Codes were again analysed and patterns among them were identified. Also, we thoroughly reviewed the literature on menstrual hygiene and associated factors to identify existing and emerging themes. Finally, we developed the following four themes for the final data analysis:

- Type of absorbent used during menstruation
- Hygiene practices during menstruation
- MHM associated morbidities
- Interventions performed to improve knowledge and practices regarding menstruation

A complete description of all the studies, including demographic details, setting, interventions, methodology and outcomes based on four themes, are presented in Additional file 1: Tables S1–S3. Quality assessment of included studies using Newcastle–Ottawa Assessment Scale shows that studies mainly were of low to moderate quality (Additional file 1: Table S4).

### Type of absorbent used during menstruation

**Fig. 2:**
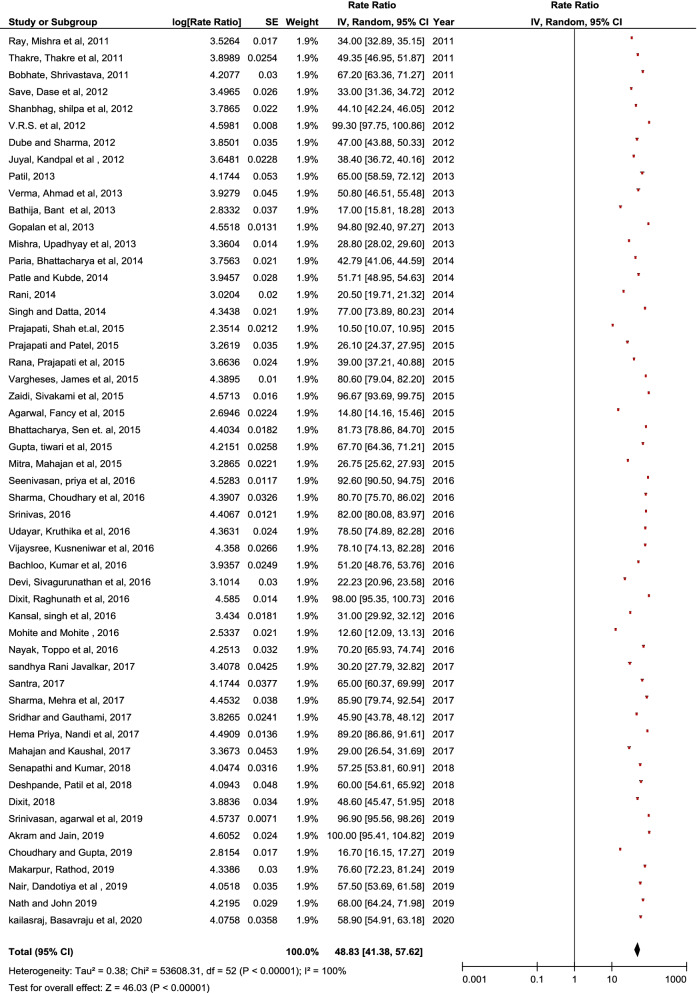
Pooled usage of sanitary pad as absorbent by Indian adolescent girls

Fifty-three studies with adequate information were included in the meta-analysis to study the use of sanitary pads by Indian adolescent girls during menstruation. Pooled data revealed a statistically significant increase in sanitary pad usage as an absorbent “(SMD = 48.83, 95% CI = 41.38–57.62, p < 0.00001)” (Fig. 2).

### Hygiene practices during menstruation

**Fig. 3:**
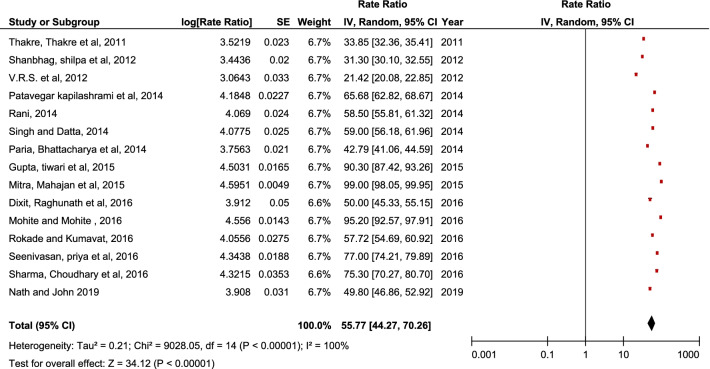
Pooled hygiene practices by Indian adolescent girls

Pooled data from fifteen studies reported a statistically significant “(SMD = 55.77, 95% CI = 44.27–70.26, p < 0.00001)” increase in perineum practices by adolescent girls during menstruation (Fig. 3). The random-effect model was used as the heterogeneity was statistically significant (p < 0.00001) and the inconsistency was too high (100%).

### MHM associated morbidities

**Fig. 4:**
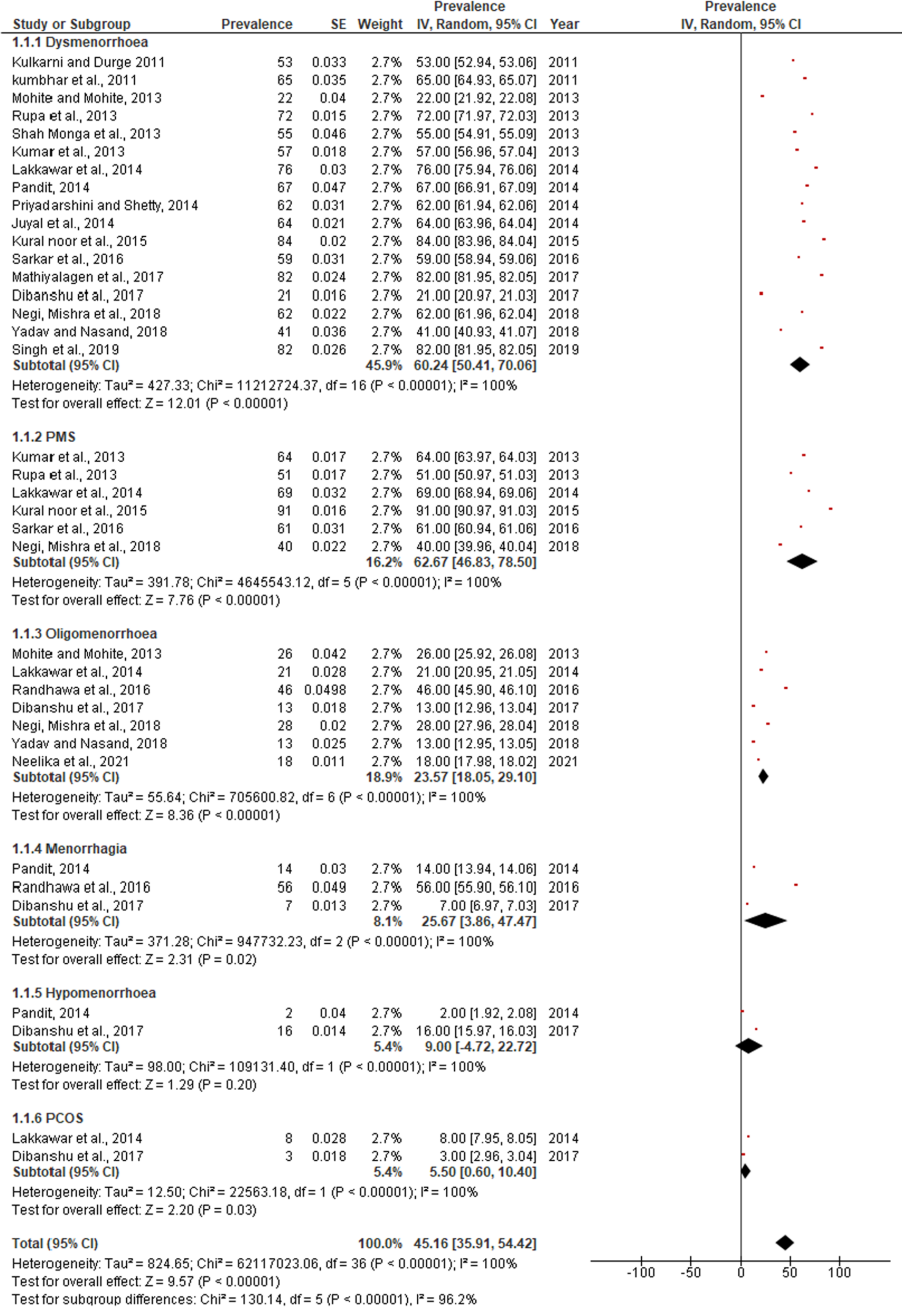
Subgroup analysis for Menstrual morbidities

Pooled data reported that most prevalent morbidities among Indian adolescent girls are dysmenorrhea “(SMD = 60.24, 95%, CI = 50.41—70.06, p < 0.0001)”, Pre-menstrual symptoms “(SMD 62.67, 95% CI = 46.83–78.50, p < 0.00001)”, Oligomenorrhea “(SMD 23.57, CI = 18.05–29.10, p < 0.00001)”, Menorrhagia “(SMD = 25.67, CI = 3.86–47.47, p < 0.00001)”, Hypomenorrhea “(SMD = 9.00, CI = 4.72–22.72, p < 0.00001)”, PCOS “(SMD = 5.50, CI = 0.60–10.40, p < 0.00001)”, and Polymenorrhea “(SMD = 4.90, CI = 1.87–12.81, p < 0.0001)”. Subgroup analysis was conducted to examine probable sources of between-study heterogeneity. The heterogeneity was statistically significant (p < 0.00001) and the inconsistency was high (100%). Figure 4 depicts a subgroup analysis of pooled data on prevalent morbidities among Indian girls. Various menstrual morbidity-associated factors, including modifiable factors, have been reported in the studies. Common modifiable associated factors include poor nutritional status, lower physical activities by girls, poor menstrual hygiene, education, and mother’s occupation. Other reported associated factors are family history, socioeconomic status, late menses, amount and duration of blood flow.

Fifteen studies have found an association between nutritional status, BMI, Junk food, meals skip during menses, dieting by girls, dietary habits, and Nutritional deficiency with menstrual morbidities. *However, one study* found no association of BMI with menstrual morbidities among Indian young girls. Five studies found that girls had a family history of menstrual morbidities are more prone to develop dysmenorrhea. Out of 21 studies, five reported that a family’s socioeconomic status could be responsible for menstrual abnormalities among adolescent girls*.* Four studies reported lower physical activity among girls with more menstrual disorders. Three studies presented the association of practices during menstruation with menstrual morbidities. Two studies reported that girls with late-onset of late menstruation and low education level have higher chances of developing menstrual problems. One study found the association with the mother’s occupation; another study has reported the association of menstrual morbidities with bleeding duration and the girl’s age. It is also found that the mother’s education has been directly associated with the menstrual problems her daughter faces.

### Interventions are performed to improve knowledge and practices regarding menstruation

**Fig. 5:**
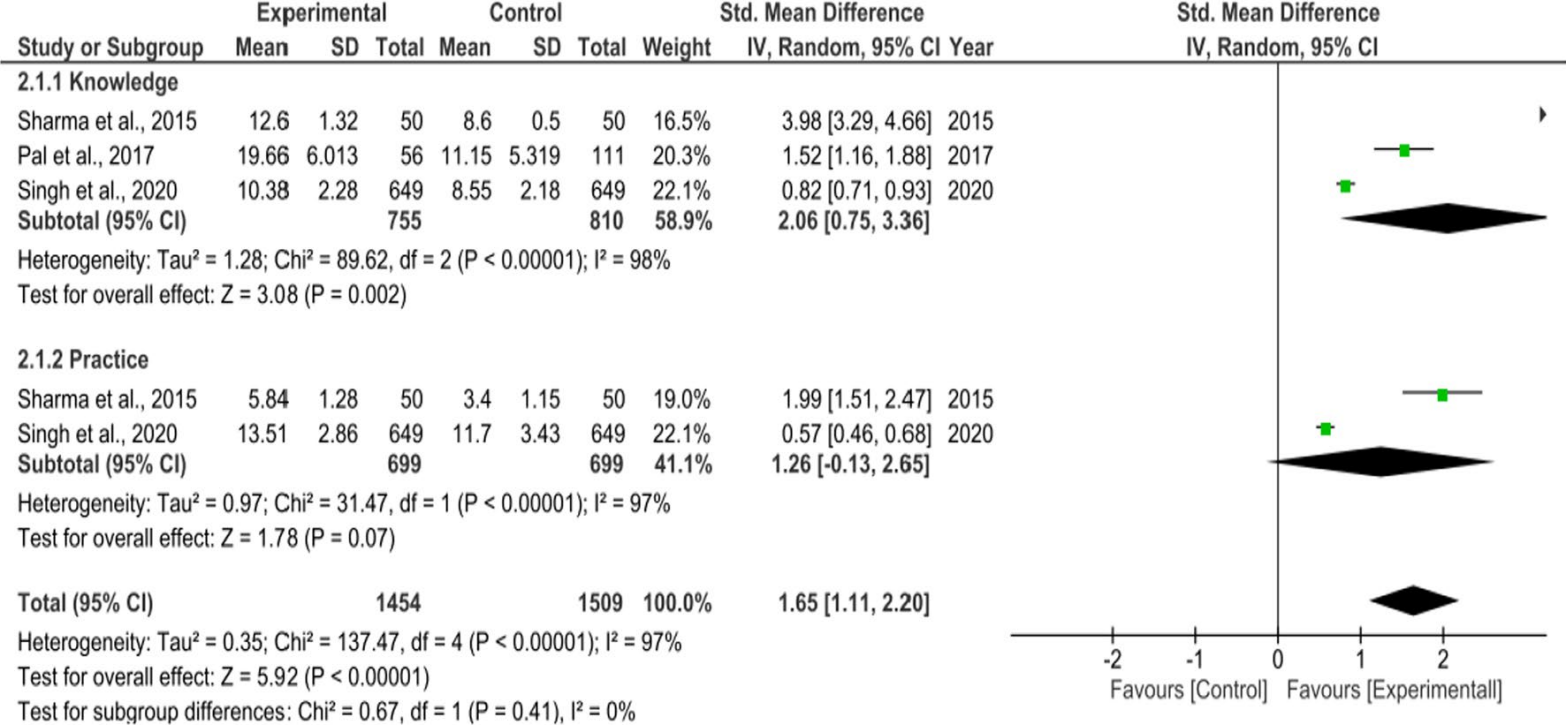
Subgroup analysis for overall changes in standardized mean difference indices for the knowledge and practice on menstruation in intervention based studies

**Fig. 6:**
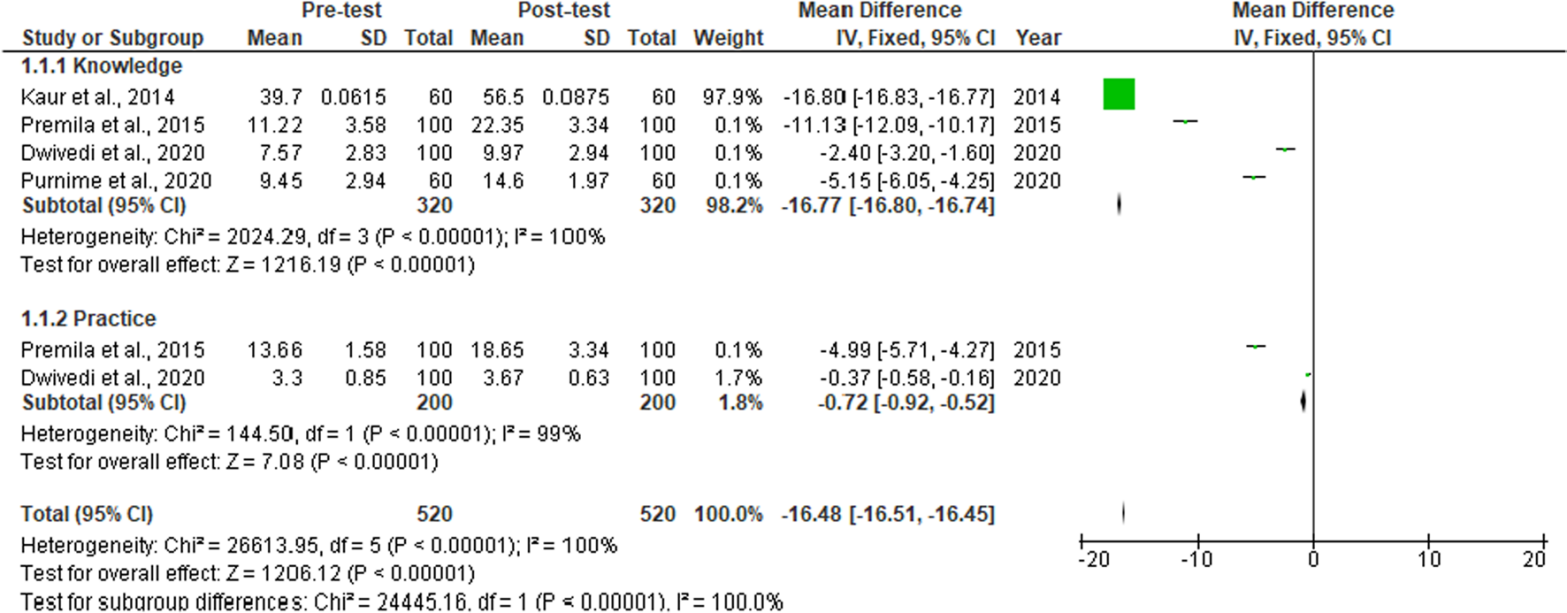
Subgroup analysis for pooled changes in standardized mean difference indices for knowledge and practice on menstruation in one group Pre-post interventional based studies

A total of fourteen interventional studies were retrieved, out of which seven were excluded due to insufficient data. Subgroup analysis was conducted to examine possible sources of between-study heterogeneity. Pooled data from three two group intervention studies revealed a statistically significant improvement in knowledge “(SMD = 2.06, 95% CI = 0.75–3.36, p < 0.00001)” and practice “(SMD = 1.26, 95% CI = 0.13–2.65, p = 0.00001)” on menstruation (Fig. 5). Pooled data from four one group pre-post interventional studies also revealed an overall improvement in knowledge “(SMD = − 16.77, 95% CI = 16.80 − 16.74, p < 0.00001)” and practice “(SMD = -0.72, 95% CI − 0.92 to − 0.52, p < 0.00001)” on menstruation among Indian adolescent girls. The heterogeneity was statistically significant (p < 0.00001) and the inconsistency was high Fig. 6.

## Discussion

The present study aimed to find studies that looked into menstrual hygiene, morbidities prevalence, and its factors, focusing on modifiable factors. Our search was susceptible to various potential biases, and it is critical to understand the type and prevalence of menstrual morbidities and their associated factors based on the result we have reported. We hoped that limiting our search and utilising broad search keywords would reduce the risk of bias in our literature selection. Hand scanning recognised articles for relevant references was added as an extra step. Given our time and resource limits, we believe we have broadened our search as far as possible. Numerous studies have been published on menstruation knowledge, awareness, and practice in low-income settings. Although each study focuses on a different context with different variables, one thing is clear: Menstrual disorders are a common problem in adolescents; at least one the menstrual morbidity is prevalent among Indian young girls, which is the source of anxiety for the patients and the families that are associated with various modifiable and non-modifiable factors [20].

### Practices by adolescent girls during menstruation

Cloths have historically been used to absorb menstrual flow; they are less expensive and less polluting, but pads are progressively replacing them, especially in urban areas. If females do not have access to water, privacy, or a drying area, cleaning and drying clothes might be challenging [21]. Commercial pads were preferred by the authors of the reviewed studies and the participants, but cost prohibits widespread usage, particularly in rural regions. Compared to a national community-based study done in 2007–2009, our pooled estimate of pad use was greater than that in a separate report from 2010, in which 12% of 1033 females sampled across India used pads [22]. Our pooled data found that more adolescents clean their perineum during menses. This is in line with findings of other studies that reported a large percentage of females to use sanitary pads, bathe every day, and cleanse their genitalia with soap and water [23, 24]. However, Only 4.6% of students in Andhra Pradesh, 11% of Haryana found washing their genitalia with soap and water during menses which could be a lack of awareness and facilities in the school.

### Menstrual disorders

In this review study, we have reported the various menstrual disorders in which dysmenorrhea has been recorded as high, premenstrual syndrome (PMS), oligomenorrhea, Mennorhagia, Polymenorrhea, Polycystic ovary syndrome (PCOS), and Hirustusmus. Many studies have also reported the same various menstrual irregularities among young girls [21, 25]. Dysmenorrhea, a medical ailment defined by intense uterine pain during menstruation presenting as the recurrent lower abdomen or pelvic discomfort that may also radiate to the back and thighs, has been recognised as the most frequent disorder [21]. There are two types of dysmenorrhea: primary dysmenorrhea and secondary dysmenorrhea. Primary dysmenorrhea occurs when there is no co-existing pathology, and secondary dysmenorrhea occurs when there is an identified and modifiable cause [26]. After dysmenorrhea, other menstrual abnormalities noted were polymenorrhea which is the condition where the gap between two consecutive cycles is 21 days, but in oligomenorrhea, it can be up to 35 days. [27] The premenstrual syndrome, also reported in various studies, is a collection of cyclic, repeating physical, emotional, and behavioural symptoms that appear during the late luteal phase of the menstrual cycle and disappear when menses begin. Symptoms range is comprehensive and affects all aspects of life (family, social, occupational). The condition for PMS diagnosis is the existence of two consecutive periods accompanied by annoying changes [27, 28]. Various factors were associated with menstrual morbidities. The main reason for morbidity prevalence could be not using the sanitary pads due to cost considerations. Other reasons included were absorbent disposal issues, lack of awareness about hygiene, and personal choice among girls.

### Common modifiable factors

Poor nutritional status, a high BMI, junk food consumption, meal skipping during menses, female dieting, decreased physical activity, low socioeconomic status and anaemia have all been identified as contributing causes to menstrual problems. Junk foods are low in micronutrients such as vitamin B6, calcium, magnesium, and potassium, which may be responsible for initiating premenstrual symptoms [14]. Another study found a link between frequent junk food consumption and irregular menstrual periods, abnormal flow, dysmenorrhea, and PMS [29]. According to Fujiwara et al., the frequency of fast food consumption was linked to dysmenorrheal [30]. To improve the menstrual health of young college girls, a study has suggested emphasising the reduction of junk food consumption and promoting healthy eating practices [31]. Also, it is essential to avoid skip of meals and diet to maintain good menstrual health. In earlier research, the lower socioeconomic level was linked to higher illness severity, morbidity, mortality, and barriers to accessing more advanced medical treatments [29, 32]. However, in the one study, the prevalence of menstruation disorders was higher in females with a middle socioeconomic class due to a sedentary lifestyle and junk food intake than in females with a low socioeconomic position owing to a sedentary lifestyle and junk food consumption [29]. Higher socioeconomic status (SES) could also be because of menstrual problems as high SES is more at high risk of consumption of fast food and a sedentary lifestyle [20].

According to the study, menorrhagia has a strong negative link with salad consumption and socioeconomic status, whereas oligomenorrhea has a favourable link with socioeconomic status. The contradictory relationship with socioeconomic level could be attributed to high socioeconomic level individuals' increased consumption of junk food, sedentary lifestyles, and lack of knowledge about healthy eating habits [29]. Good nutrition and a healthy lifestyle induce puberty earlier. As menstruation is the last process of puberty, it delays adolescence with stress, poor nutrition, and an unhealthy lifestyle [33, 34]. Physical activity and menstrual irregularities have been found to have a strong link. Compared to students who did not exercise regularly for more than 3 days a week, regular students had fewer menstrual irregularities in cycle length, flow, dysmenorrhea, and PMS. Regular physical activity helps maintain optimal body weight, enhance insulin sensitivity, enhance BMR, and release endorphins, which aid in menstrual cycle regularisation, PCOS and hypothyroidism improvement, PMS reduction, and overall well-being [14, 35].

Adolescent gynecologic problems are unique in the spectrum of gynecologic illnesses of all ages, as 75% of women suffer from menstrual issues. A statistically significant link between a girl’s educational level and reproductive morbidity has been reported. As one's level of education rises, so does one’s age in years, and as one’s age rises, so does reproductive morbidity [36]. Girls with a lower educational level may be unwilling to discuss reproductive morbidity. This could explain the current study’s findings. It is suggested that an aware girl and a mother continue with girl education could improve the family’s well-being and quality of life. Poor menstrual hygiene practices are closely linked to reproductive tract morbidities, damaging a woman’s life. According to research, a large information gap exists among adolescent girls in terms of prior awareness of menstruation and menstrual cleanliness, which impacts menstrual practices and associated gynaecological morbidities [37]. Different cultures in India have different types of limitations during menstruation, which could be a factor in the lack of compliance with proper menstrual hygiene [38, 39]. PCOS was linked to a higher incidence of diabetes mellitus and hypothyroidism in the family. According to previous research, the likelihood of discovering a metabolic problem in the families of PCOS patients is 2.7 times higher than in the control group [40]. As a result, PCOS is more common in girls whose parents and grandparents had metabolic abnormalities than in girls whose parents and grandparents did not. Hypertension is three times more common in women with PCOS than in those without the condition, according to previous research [20].

With intervention, the post-test and experimental groups significantly improved menstrual hygiene compared to the pre-test and control groups. This study is backed by various studies that found a need for health education and behaviour change programmes in this area [41–43]. This type of intervention in schools can eradicate preconceptions, prejudices, and improper familial behaviours. So imparting the proper knowledge, changing attitudes and practices, and reducing the likelihood of developing morbidities like RTI [44]. A study conducted in Egypt also found that a menstrual education programme for first and second-year girls at a secondary school was adequate and that the programme needed to be expanded to elementary, preparatory, and other secondary schools [45]. A similar study by Chang et al. on primary school girls in grades 5 and 6 found that educational programmes in schools for students and their parents were effective [43]. Another study conducted in Bangladesh found a 31.4 per cent improvement in follow-up knowledge scores, identical to the current study but with only one intervention group [46].

By examining replies and gauging retention after the intervention, our study offers evidence to support the efficacy of education training in producing a lasting influence on knowledge levels. Furthermore, few studies have looked at changes in views immediately after intervention and several months later. The importance of family life education in the school health programme has been recognised. We can increase knowledge by including themes on specific physiological aspects of menstruation and pregnancy in health education programmes offered by health professionals for adolescent girls [47].

We recommend that public health programmes strengthen menstrual hygiene management and associated factors. Evidence-based research, particularly research targeting the most underserved to assess the actual impact and outcomes of programmes targeting MHM should be conducted in the country. Support for learning from the implementation of government programmes and policies to share across country governments, longitudinal research to measure relevant impact and outcomes; increased investment in the evidence base for addressing MHM in schools, particularly research targeting the most underserved; and a better understanding of costs and effectiveness, as well as the benefits of comprehensive, cross-sectoral addressing MHM in schools are among the key recommendations for actions that will advance the agenda. All of these efforts will help the young girls to become aware of and comfortable with their menstrual cycle and will be able to manage their periods in a pleasant, safe, and dignified manner.

### Implications

The results of this study can be helpful in future studies to prevent and treat menstrual disorders such as dysmenorrhea, oligomenorrhea, and premenstrual syndrome to promote menstrual health by modifying lifestyle and improving the quality of life of young Indian girls. Furthermore, this study suggests the need for research on associated factors of specific menstrual problems and the role of comprehensive intervention on menstruation among adolescent girls.

## Conclusion

Menstruation-related problems are widespread among adolescent girls in India. Studies show that menarche results in feelings of stress, anxiety, depression, and anger. Our findings estimate that most Indian adolescent girls began menarche unaware of the reason, with very few knowing the source of bleeding. Learning about menstrual hygiene and health is an essential aspect of adolescent girls’ health education to continue working and maintaining hygienic habits. Infections of the reproductive system and their repercussions can be avoided with better awareness and safe menstruation practices. The ideal menstrual health education programme would teach students to consider the connections between knowledge, behaviour, and improved human health. It would also assist in the improvement of maternal health. There is a need to address the menstrual morbidities in young girls’ initial stages of life. Therefore workshops, adding a chapter to some course’s literature focusing on improving the lifestyle and associated modifiable factors with raising girls’ general information about the following: physiology of menstruation, the relationship between hormonal changes, symptoms and menstrual disorders and their associated factors.

### Strengths and limitations

There is the possibility of selective reporting bias or publication prejudice in any review. Our review found many outcomes, ranging from substantially positive associations to the inverse. We attempted to broaden our review to include these studies, given the time and resources available. However, this was not possible due to time and resource constraints. With these constraints in mind, we have concluded that most studies on menstrual hygiene practices, morbidities, and their associated factors focus on determining the prevalence of exposure. We conducted a systematic search for articles and included research based on explicitly specified criteria to reduce selection bias, which strengthened this review.

Furthermore, between-study high heterogeneity is observed in the present review as depicted by I^2^ statistic. This may be due to different methodologies and study settings. We developed four themes to analyse and interpret our results and conducted subgroup analysis by type of morbidity and study design.

## Supplementary Information

**Additional file 1. Table 1.** Morbidities and Associated factors. **Table 2.** Use of absorbent and perineum cleaning during menses. **Table 3.** Intervention based studies on menstruation. **Table 4.** Quality assessment of included studies by using Newcastle – Ottawa Assessment Scale

## Data Availability

All data relevant to the study are included in the article and additional sheets. No additional data is available.

## Abbreviations

BMI: Body mass index
BMR: Basic metabolic rate
LMICs: Low and middle-income countries
MHM: Menstrual hygiene management
PCOS: Polycystic ovary syndrome
PICO: Population intervention comparison of outcome
PMS: Premenstrual syndrome
PRISMA: Preferred Reporting Items for Systematic Reviews and Meta-Analyses
SES: Socioeconomic status
SMD: Standardized mean difference
UTI: Urinary tract infections
