# Supplementary material for: Menstrual hygiene practices and associated factors among Indian adolescent girls: a meta-analysis
Source: Reprod Health. 2022 Jun 23;19:148. doi: 10.1186/s12978-022-01453-3 (PMC9229495; doi:10.1186/s12978-022-01453-3)
Supplement: Supplementary file 1 — Additional file 1. Table 1. Morbidities and Associated factors. Table 2. Use of absorbent and perineum cleaning during menses. Table 3. Intervention based studies on menstruation. Table 4. Quality assessment of included studies by using Newcastle – Ottawa Assessment Scale [file 12978_2022_1453_MOESM1_ESM.docx]

**Supplementary files**

**Menstrual Hygiene Practices and Associated Factors Among Indian Adolescent Girls: A Meta-analysis**

**Table 1 – Morbidities and Associated factors**

| **Author (Year)** | **State** | **Sample size** | **Age of participants**  **(Years)** | **Setting** | **Duration** | **Morbidity (%)** | **Associated Factors** |
| --- | --- | --- | --- | --- | --- | --- | --- |
| Kumbhar, Reddy et al. (2013) | Andhra Pradesh | 183 | 14-19 | School and college | 5 months | Dysmenorrhea - 65% | -Family History |
| Kumar, Goel et al. (2013) | Chandigarh | 744 | 13-19 | Rural, Urban and Slum strata | 1 Year | Dysmenorrhea  - 57.7%  PMS -64.1% | -Socio-economic status |
| Mohite and Mohite (2013) | Maharashtra | 107 | 17-20 | College | 2 months | Dysmenorrhea - 22.4%  Irregular MC – 26.1% | -Poor nutritional status  -Anaemia  -Low SES |
| Rupa Vani, Veena et al. (2013) | Pondicherry | 861 | 13-19 | School | - | Dysmenorrhea - 72.8% PMS– 51.2 % | - BMI  - Junk food  - Dieting  - Physical activity |
| Shah, Monga et al. (2013) | Gujarat | 116 | 18-21 | Nursing College | 6 months | Dysmenorrhea- 55% | -No association with BMI and ovarian volume |
| Lakkawar, Jayavani et al. (2014) | Pondicherry | 200 | 17-22 | Medical College | - | Polymenorrhea – 8%  Oligomenorrhea – 21%  Dysmenorrhea- 76%  PMS- 69% | -Overweight (BMI)  -Junk food  - Dieting  -Physical activity |
| Juyal, Kandpal et al. (2014) | Dehradun | 485 | 15-18 | Inter college | - | Dysmenorrhea- 64.9%  RTI- 18.8% | - Hygiene practice |
| Priyadarshini and Shettyet al. (2014) | Karnataka | 233 | 12-17 | School | 2 months | Dysmenorrhea- 62.7% | -Family history |
| Pandit (2014) | Maharashtra | 100 | 11-15 | School | - | Menorrhagia- 14%  Hypomenorrhea- 2%  Dysmenorrhoea- 67%  PMS-84% | -Nutritional status (BMI) |
| Kural, Noor et al. (2015) | Madhya Pradesh | 310 | 18-25 | Medical College | 3 months | Dysmenorrhea- 84.2%  PMS – 91% | -Bleeding duration (more than 5 days)  -Family history |
| Joshi, Kural et al. (2015) | Madhya Pradesh | 310 | 18-25 | Medical College | 3 months | Dysmenorrhea - 84.2%  PMS – 91% | -Meal skip |
| Sarkar, Mandal et al. (2016) | West Bengal | 244 | 13-21 | School | - | Dysmenorrhea- 59.4%  PMS – 61.5% | -Occupation of mother |
| Randhawa, Mahajana et al. (2016) | Punjab | 100 | 17-33 | College | 3 Months | Menorrhagia- 56.25% Menorrhagia - 44.68% Oligomenorrhea - 46.81% | - Nutritional status (Junk food)  -Socio-economic status |
| Dibyanshu, N.B. Kasturwar et al. (2017) | Nagpur | 600 | 10-19 | Rural Health Centre | 1.6 year | Dysmenorrhea - 21%, Oligomenorrhea - 13% Menorrhagia- 7%, Polymenorrhea - 3% Hypomenorrhea - 16 % | -Age of girl (late adolescent)  - Mother education  - Girl education  - Anaemia |
| Mathiyalagen,, Peramasamy et al. (2017) | Puducherry | 242 | 12-18 | School | 1 month | Dysmenorrhea - 82.2% | -Poor menstrual hygiene practices |
| Kulkarni and Durge (2011) | Nagpur | 224 | 10-19 | Slum area | 8 months | Dysmenorrhea - 53.6% | - Education  - Age of girl |
| Yadav and Masand (2018) | Jaipur | 180 | 10-19 | Outpatient of Obstetrics and Gynecology outpatient | 1.5 years | Dysmenorrhea - 41.66%  Heavy MC bleeding-. 25%  Irregular MC bleeding - 13.33% | -Poor menstrual hygiene  -Nutritional deficiency  -Anemia |
| Negi, Mishra et al. (2018) | Garhwal | 470 | 13-19 | School | - | Dysmenorrhea -62.75%  PMS -40.42%  Irregular cycle - 28.72% | -Dietary habits  -Lack of physical activities |
| Desai, Tiwari et al. (2018) | Gujarat | 881 | 13-18 | School | 7 months | PCOS-13.54% | -Obesity (BMI)  -Socio-economic status  - Family history |
| Singh, Rajoura et al. (2019) | Delhi | 210 | 11-19 | School | - | Dysmenorrhea - 82.4%. | -Nutritional status ( low BMI) |
| Neelika Tripathi et al. (2021) | Agra | 1200 | 18-30 | College | 1 year | Menstrual irregularity – 18.30%  Hirsutism-8.40% | -Socioeconomic status  -Dietary habits  -Lack of physical activity  -Obesity  -Family history |

**Table 2 – Use of absorbent and perineum cleaning during menses**

| **Author (Year)** | **State** | **Sample size** | **Inclusion and exclusion criteria** | **Setting** | **Data collection tool** | **Result** |
| --- | --- | --- | --- | --- | --- | --- |
| Makarpur, Rathod et al. (2019) | Karnataka | 150 | Age 14-16 years and not suffering from any systemic disease | School | Questionnaire | -76.6% used Sanitary pads |
| Ray, Mishra et al. (2011) | West Bengal | 700 | Attained menarche at least 2 years before the date of interview. | Rural areas and municipal wards in urban area | Interview | -Urban girls have better menstrual hygiene practices (β = 0.343, p < 0.01) than rural girls. |
| Gopalan et al. (2020) | Pondicherry | 325 | Age 12-15 years | School | Questionnaire | 94.8% girls said they used sanitary napkins as absorbent during menstruation. |
| Gosavi, Almale et al. (2015) | Nashik, Maharashtra | 60 | Girls from migrant’s population (Dera) | Corporation area | Interview | All participants were used cloths during menstruation and they disposed of those clothes in open landfill after its use. |
| Bathija, Bant et al. (2013) | Karnataka | 100 | Age 15-45 years, women who had not attained menarche, or/and menopause | Urban slums | Questionnaire | 17% participants were using sanitary pads. |
| Singh and Datta (2014) | Pondicherry | 371 | Girls who attained menarche in the age group 11-18 years | School | Focus Group discussions and Questionnaire | Sanitary pads were used by 77% girls and 59% had habit of washing genitalia with soap and water during menses. |
| Paria, Bhattacharyya et al. (2014) | West Bengal | 541 | Girls in age group 13–18 years. | Urban and rural area | Questionnaire | 45.4 were using sanitary pads and 42.79 % were cleaning genitals during menstruation regularly. |
| Dube and Sharma (2012) | Rajasthan | 200 | Girls in age group 15-19 years. | School | Questionnaire | 47% were using branded sanitary napkins. |
| Akram and Jain (2019) | Tamil nadu | 149 | Girls of class 8 to class10. | School | Questionnaire | 100% were using sanitary pad as absorbent during menstruation. |
| Choudhary and Gupta (2019) | Rajasthan | 450 | Girls of 8-12 class who had attended their menarche. | School | Questionnaire | 16.7% girls were using sanitary pads during menstruation as absorbent. |
| Misra, Upadhyay et al. (2013) | Haryana | 995 | Girls in age group 15-45 years. | Rural | Interview using questionnaire. | Only 28.8% of women were using sanitary napkins. |
| Rana, Prajapati et al. (2015) | Gujarat | 400 | Girls of 14 to 17 years of age | School | Interview using questionnaire. | 39% used sanitary pad during last menstruation. |
| Verma, Ahmad et al. (2013) | Varanasi | 120 | 9 to 12 class girls. | School | Questionnaire | Only 50.8% girls used sanitary pads, and 49.16% girls used old cloth. |
| Varghese, James et al. (2015) | Chennai, Tamil Nadu | 1522 | Girls aged 14-17 years of standards IX to XI who had attained menarche. | School | Questionnaire | 80.6% were using sanitary pads. |
| V.R.S. et al. (2012) | Coimbatore, tamil Nadu | 144 | Girls in age group: 13 to 19 years | School | Interview | 99.30% of the girls has chosen napkin as their absorbent and 21.42 % cleaned perineum during menstruation. |
| Shanbhag, Shilpa et al. (2012) | Bangalore | 506 | Girls studying in 8, 9 and 10th class. | School | Questionnaire | Only 44.1% used sanitary pad during the menstrual cycles and only 31.3% used soap and water to clean their genital organs. |
| Singh, A.et al, (2013) | Uttarakhand | 200 | Girls of khirshu village, Garhwal. | Rural | Questionnaire | 38% girls used sanitary pads and 62% used cloth during menses. |
| Thakre, Thakre et al. (2011) | Nagpur | 387 | Girls studying in 8 and 9 class | School | Questionnaire | Sanitary pads were used by 49.35% of the selected girls while 33.85% practice perineum cleaning. |
| Srinivas (2016) | Pondicherry | 1000 | Girls studying in 8 to 12 class who had attained menarche. | School | Questionnaire | 82% were used sanitary napkins, 12% cotton and 6% cloth as absorbent during menses. |
| Nair, Dandotiya et al. (2019) | Bhopal | 200 | Secondary school girls (Classes IX, X, XI, and XII). | School | Questionnaire | Only 57.5 % used sanitary pads. |
| Bobhate and Shrivastava (2011) | Mumbai | 242 | Girls from 10 to 19 years of age who had attained menarche. | Urban slum | Questionnaire | 32.8% subjects had unsatisfactory menstrual hygienic practices. |
| Patle and Kubde (2014) | Nagpur | 310 | Girls in age group 10-19 years. | College | Questionnaire | 62.03% of urban girls and 43.4% of rural girls were using commercially available sanitary pads. |
| Rani (2014) | Andhra Pradesh | 400 | Girls in age group 15-25 years. | Rural | Interview via questionnaire | 58.5% clean the perineum during menstruation after each and every urination and or defecation. 77.5% used clean old cotton cloth as perineal pads, whereas about one-fifth of them (20.5%) using sanitary napkins. |
| Zaidi, Sivakami et al. (2015) | Tamil nadu | 150 | Girls who attained menarche | School | Questionnaire | 96.67% girls were using sanitary pads as absorbent. |
| Patil (2013) | Karnataka | 80 | Girls in age group 11-16 years who attained menarche. | Medical | Questionnaire | 65% were using sanitary napkins. |
| Salve, Dase et al. (2012) | Aurangabad | 321 | Girls studying in 8th to 10th standard | School | Questionnaire | Sanitary napkins use was more in urban girls 56(60%) compared to rural girls 6 (06%) whereas homemade sanitary napkins were used by 87(94%) rural girls & 38 (40%) urban girls. |
| Hema Priya, Nandi et al. (2017) | Pondicherry | 528 | Girls in age group 10 to 19 years. | Rural | Questionnaire | 89.2% of the adolescent girls were using sanitary pads; fresh and reusable cloths were used by 6.6% and 4.2%, respectively. |
| Sharma, Mehra et al. (2017) | Delhi | 85 | Girls in age group 10-19 years | Urban | Interview | 85.9% girls used sanitary pads during menstruation. |
| Juyal, Kandpal et al. (2012) | Uttarakhand | 453 | Girls studying in 9 to 12 | College | Interview via questionnaire | 38.4% adolescent girl was used sanitary napkins and 30% used cloth as absorbent. |
| Dixit, Raghunath et al.  (2016) | Madhya Pradesh | 100 | Girls in age group 12-16 years. | School | Questionnaire | For genital cleaning 44% girls used both soap and water with 60% girls from government school and 28% from private school and 56% girls used plain water. Sanitary pads were used among 98% girls. |
| Deshpande, patil et al. (2018) | Maharashtra | 100 | Girls in age group 10 to19 years | House-to-House survey | Questionnaire | About 60% girls used sanitary pad and the rest used cloth pieces. |
| Mohite and Mohite (2016) | Maharashtra | 230 | Girls who had attained menarche, and unmarried. | Slum area | Interview via questionnaire | 12.6% were practiced disposable absorbent sanitary napkins whereas 87.3% practiced reusable cloth materials while 95.2% maintained genitals cleaning. |
| Prajapati and Patel (2015) | Gandhinagar | 155 | Unmarried, non pregnant, non- lactating females in age group 10 -19 years. | Anganwadi | Questionnaire | Sanitary pad was used by 26.1% girls. Around 30% girls were not using sanitary pad because of cost. |
| Udayar, Kruthika et al. (2016) | Andhra Pradesh | 293 | Girls in age group 10-19 years | Rural | Questionnaire | Sanitary pads usage during menstruation was seen in 78.5%. |
| Nayak, Toppo et al. (2016) | Madhya Pradesh | 200 | Girls in age group 10–19 years. | Urban | Questionnaire | A total of 70.2% of the girls used sanitary pads but the rest used cloth and the practice of reusing without washing properly was seen commonly among girls. |
| Kansal, Singh et al. (2016) | Varanasi | 590 | Girls in age group 15-19 years | Rural | Semi- structured interview | Only 31% respondents were using sanitary pads during menstruation. |
| Mahajan and Kaushal (2017) | Himachal Pradesh | 100 | Girls from class 9th to 12th who attained menarche. | School | Questionnaire | 29% had adequate knowledge about menstrual hygiene, 71% had inadequate knowledge about menstrual hygiene. |
| Dixit (2018) | Etawah | 212 | Girls in age group 10-19 years and have attained menarche. | College | Questionnaire | Only 48.6% girls used sanitary pads as the absorbent material. |
| Nath and John (2019) | Tamil Nadu | 250 | Girls in age group 10-19 years who have started menstruating and have had three cycles. | Rural | Interview by using Questionnaire | 68% girls used commercially available sanitary pad as menstrual absorbents and 21.2% use both sanitary pad and cloth. - 49.8% were clean their perineum during menstruation. |
| Sandhya Rani Javalkar (2017) | Karnataka | 116 | Girls in age group 11 and 18 years. | - | Interview by using Questionnaire | Only 30.2% of girls used sanitary pads. |
| Bhattacharya, Sen et al. (2015) | Kolkata | 460 | Girl students of class X and XI in age group 14-16years. | - | Questionnaire | 81.73% girls used only sanitary napkin. |
| Prajapati, Shah et al. (2015) | Gujarat | 200 | Girls who had attained menarche studying in class 8 to 12. | Rural | Questionnaire | 10.5% respondents used only sanitary napkin during menstruation. |
| Devi, Sivagurunathan et al. (2016) | Tamil Nadu | 180 | Girls in age group 11-16 years who attained menarche. | School | - | 35.55% girls reported use of old cloth for protection during menstruation. |
| Vijaysree, Kusneniwar et al. (2016) | Telangana | 242 | Girls studying in 8th, 9th & 10th standards who attained menarche. | School | - | 21.9% do not use sanitary pads. |
| Senapathi and Kumar (2018) | Karnataka | 244 (Rural -132 and Urban - 112 ) | Girls in age group 13–18 years, studying in 9 and 10 standard that had attained menarche. | College | - | 49.24% of the girls in the rural school and 65.17% of the girls in urban school were using sanitary pads. |
| Bachloo, Kumar et al. (2016) | Haryana | 400 | Girls in age group 13-19 years. | School | - | 51.2% of the adolescents use sanitary pad and 40.6% use old cloth while rest uses both. |
| Rokade and Kumavat (2016) | Maharashtra | 324( Slum -200, Non-slum -124) | Girls who had attained menarche and not physically or mentally handicapped | College | House to house survey via questionnaire. | 57.72 were cleaned genitals during menstruation. |
| Gupta, Tiwari et al. (2015) | Indore, Madhya Pradesh | 330 (Urban- 279 Rural - 71) | 15-45 years | College | Interview via Questionnaire | 67.7% used sanitary pads while rest used cloths.  - The cleaning of external genitalia daily was practiced by 90.3% of urban living participants where in those living in rural areas were 83.09%. |
| Mitra, Mahajan et al. (2015) | Gujarat | 400 | Girls who attained menarche. | Anganwadi centre | Questionnaire | 73.25% girls were using clothes while 26.75% were using sanitary napkin. - 99%girls practiced cleaning of genitals with water. |
| Agarwal, Fancy et al. (2017) | Sabarkantha District, Gujarat | 250 | 8th to 12th standard girls who had attained menarche. | Rural government school | Interview via Questionnaire | 14.8% respondents used only sanitary napkin during menstruation. |
| Sridhar and Gauthami (2017) | Telangana | 425 | Girls in the age of 10-19 yrs including married, antenatal and postnatal subjects. | Urban | Questionnaire | 45.9% were using sanitary napkins as absorbent. |
| Seenivasan, Priya et al. (2016) | Chennai | 500 | Girls who have attained menarche. | School | Questionnaire | Sanitary pads were used by 92.6%, other absorbents being new cloth (5%) and old cloth (0.6%).  - Washing their genitals more than two times a day was noted in 77%. |
| Kailasraj, Basavaraju et al. (2020) | Bangalore | 190 | Girls who had attained menarche. | School | Questionnaire | 58.9% were using sanitary pads during menstruation and 46.8% changes absorbents twice a day. |
| Santra (2017) | West Bengal | 160 | 15-45 years old girls | Slum | Questionnaire | 65% women used only sanitary pad and 30% used only cloth pieces where as 5% used both pad and cloth piece. |
| Sharma, Choudhary et al. (2016) | Haryana | 150 | Girls in age group 11-16 years who had attained menarche. | School | Questionnaire | 80.7% girls used sanitary napkins during menstruation. - Only 75.3% girls washed their external genitalia regularly. |
| Srinivasan, Agarwal et al. (2019) | Bangalore | 758 | Male and female students studying in a degree college. | College | Questionnaire | 384 (99.5%) were aware of sanitary napkins, 165 (42.7%) of menstrual cup, 12 (3.1%) of tampon and 254 (65.8%) of reusable cloth, however majority (96.9%) preferred use of sanitary napkins. |

**Table 3 –Intervention based studies on menstruation**

| **Author (Year)** | **State of India** | **Inclusion criteria** | **Sample size** | **Setting** | **Intervention** | **Control group - Pre intervention** | **Experimental - Pre Intervention** | **Control group - Post Intervention** | **Experimental - Post Intervention** |
| --- | --- | --- | --- | --- | --- | --- | --- | --- | --- |
| Singh, gupta et al. (2020) | Haryana | 9th to 12th standard and excluded who were seriously ill such as malaria, severe anemia, high-grade fever etc. | 649 | Rural health center, | Education session  1 session | Knowledge - 4.94±1.83 practice- 8.28±3.94 | Knowledge - 4.99±1.82  Practice - 8.34±3.96 | Knowledge - 8.55±2.18 11.70±3.43 | Knowledge - 10.38±2.28 Practice- 13.51±2.86 |
| Kaur, Vadivukkarassi et al. (2014) | Punjab | Age group of 10-19 years | 60 | School | Teaching programme for  40 minutes | Knowledge - 39.7 | - | - | Knowledge - 56.5 |
| Dwivedi, Sharma et al. (2020) | Rajasthan | Aged 11–19 years, studying in class 6th to 12th. | 100 | School | Education by trained peer group. | Knowledge - 7.57±2.83 Attitude-2.38±1.14  Practice- 3.30±0.85 | - | - | Knowledge- 9.97±2.94 Attitude - 3.33±0.948 Practice - 3.67±0.63 |
| Sharma, Negi et al. (2015) | Dehradun | Age group of 11 to 17 years and had minimum one menstrual period in last 3 months. | 50 | School | Education session  1 session | Knowledge- M- 8.02, SD- 0.50 Practice - M-3.24, SD- 1.15 | Knowledge- M- 8.04, SD - 1.32 - Practice- M-3.52, SD- 1.28 | Knowledge- M- 8.6, SD- 0.50 - Practice : M- 3.4, SD- 1.15 | Knowledge: M- 12.6, SD - 1.32 - Practice; M- 5.84, SD-1.28 |
| Pal Ahmad et al. (2017) | Kolkata | Students of class V-VIII, who have achieved menarche. | Control- 111 Intervention - 56 | School | Education sessions  2 sessions in a week for 6 months | Baseline - M- 10.76, SD-3.788 | Baseline : M-11.49, SD- 4.106 | 6 months: M-10.76, SD - 5.177 9 months : M-11.15, SD- 5.319 | 6 months : M-22.10, SD - 6.227 9 months : M-19.66, SD - 6.013 |
| Premila Ganesh et al (2015) | Puducherry | 9-10 class girls who attained menarche. | 100 | School | Education session for  1 hour | Knowledge of menstrual hygiene : Mean - 11.22, SD- 3.58 Practice: Mean - 13.66, SD-1.58 | - | - | Knowledge of menstrual hygiene : Mean - 22.35, SD- 3.34 Practice: Mean -18.65 , SD-3.34 |
| Purnima Sahoo et al, (2020) | Odisha | Girls  of KISS school. | 60 | School of Bhubaneshwar | Education session   - | Menstrual hygiene Knowledge Mean - 9.45, SD- 2.94 | - | - | Menstrual hygiene Knowledge Mean - 14.6, SD- 1.97 |

**Table 4 – Quality assessment of included studies by using Newcastle – Ottawa Assessment Scale**

| **QUALITY ASSESSMENT OF CROSS – SECTIONAL STUDIES** | | | | | | | | | | | | | | |
| --- | --- | --- | --- | --- | --- | --- | --- | --- | --- | --- | --- | --- | --- | --- |
|  | **SELECTION** | | | | | | **COMPARABILITY** | | **OUTCOME** | | | |  | |
| **Author, Year** | **Representativeness of the sample:**  A) Truly representative of the average in the target population. * (all subjects or random sampling)  B) Somewhat representative of the average in the target population. * (non-random sampling)  C) Selected group of users.  D) No description of the sampling strategy. | **Sample size:**  A) Justified and satisfactory. *  B) Not justified. | **Non-respondents:**  A) Comparability between respondents and non-respondents characteristics is established, and the response rate is satisfactory. *  B) The response rate is unsatisfactory, or the comparability between respondents and non-respondents is unsatisfactory.  C) No description of the response rate or the characteristics of the responders and the non-responders. | | **Ascertainment of the exposure (risk factor):**  A) Validated measurement tool. **  B) Non-validated measurement tool, but the tool is available or described.*  C) No description of the measurement tool. | | **The subjects in different outcome groups are comparable, based on the study design or analysis. Confounding factors are controlled.**  A) The study controls for the most important factor (select one). *  B) The study control for any additional factor. * | | **Assessment of the outcome:**  A) Independent blind assessment. **  B) Record linkage. **  C) Self report. *  D) No description. | | **Statistical test:**  A) The statistical test used to analyze the data is clearly described and appropriate, and the measurement of the association is presented, including confidence intervals and the probability level (p value). *  B) The statistical test is not appropriate, not described or incomplete. | | **Number of stars** | |
| Kumbhar, Reddy et al. (2013) | B | B | C | | A | | A | | C | | A | | 6 | |
| Kumar, Goel et al. (2013) | A | A | B | | A | | A | | B | | A | | 8 | |
| Mohite and Mohite (2013) | B | B | C | | A | | A | | C | | A | | 6 | |
| Rupa Vani, Veena et al. (2013) | C | B | A | | C | | B | | B | | A | | 5 | |
| Shah, Monga et al. (2013) | A | A | C | | C | | A | | C | | A | | 5 | |
| Lakkawar, Jayavani et al. (2014) | B | B | C | | B | | B | | C | | A | | 5 | |
| Juyal, Kandpal et al. (2014) | A | A | A | | A | | A | | C | | A | | 8 | |
| Priyadarshini and  Shetty (2014) | A | B | C | | A | | A | | B | | A | | 7 | |
| Pandit (2014) | C | B | C | | A | | B | | C | | A | | 5 | |
| Kural, Noor et al. (2015) | C | B | C | | B | | A | | B | | A | | 5 | |
| Joshi, Kural et al. (2015) | C | B | C | | B | | A | | B | | A | | 5 | |
| Sarkar, Mandal et al. (2016) | A | B | C | | A | | A | | C | | A | | 5 | |
| Randhawa, Mahajana et al. (2016) | B | B | C | | C | | A | | C | | A | | 4 | |
| Dibyanshu, N.B. Kasturwar et al. (2017) | A | A | A | | A | | B | | C | | A | | 8 | |
| Mathiyalagen,, Peramasamy et al. (2017) | A | A | C | | A | | A | | A | | A | | 7 | |
| Kulkarni and Durge (2011) | A | B | C | | A | | B | | A | | A | | 7 | |
| Yadav and Masand (2018) | B | A | C | | B | | A | | C | | B | | 5 | |
| Negi, Mishra et al. (2018) | B | B | C | | B | | A | | C | | A | | 5 | |
| Desai, Tiwari et al. (2018) | B | A | A | | A | | A | | C | | A | | 8 | |
| Singh, Rajoura et al. (2019) | B | B | C | | B | | A | | C | | A | | 5 | |
| Neelika Tripathi et al. (2021) | A | A | C | | B | | A | | A | | A | | 7 | |
| Makarpur, Rathod et al. (2019) | D | B | C | | A | | A | | C | | B | | 4 | |
| Ray, Mishra et al. (2010) | B | A | C | | A | | A | | C | | A | | 7 | |
| Gopalan et al. (2020) | B | B | C | | B | | A | | C | | A | | 5 | |
| Gosavi, Almale et al. (2015) | A | A | C | | B | | A | | C | | B | | 5 | |
| Bathija, Bant et al. (2013) | D | A | C | | A | | A | | C | | A | | 6 | |
| Singh and Datta (2014) | B | A | C | | A | | A | | C | | B | | 6 | |
| Paria, Bhattacharyya et al. (2014) | A | A | A | | A | | B | | C | | A | | 8 | |
| Dube and Sharma (2012) | B | B | C | | B | | A | | C | | A | | 5 | |
| Akram and Jain (2019) | A | A | C | | A | | A | | C | | A | | 7 | |
| Choudhary and Gupta (2019) | B | A | C | | A | | A | | C | | A | | 7 | |
| Misra, Upadhyay et al. (2013) | A | A | C | | A | | A | | C | | A | | 7 | |
| Rana, Prajapati et al. (2015) | B | A | C | | A | | A | | C | | B | | 6 | |
| Verma, Ahmad et al. (2013) | C | B | C | | A | | A | | C | | A | | 5 | |
| Varghese, James et al. (2015) | B | B | C | | A | | A | | C | | A | | 6 | |
| V.R.S. et al. (2012) | A | A | C | | A | | A | | C | | A | | 7 | |
| Shanbhag, Shilpa et al. (2012) | C | B | C | | B | | A | | C | | A | | 4 | |
| Singh , A et al, (2013) | B | A | C | | A | | A | | C | | A | | 5 | |
| Thakre, Thakre et al. (2011) | B | A | C | | A | | A | | C | | A | | 7 | |
| Srinivas (2016) | C | B | C | | B | | A | | C | | A | | 4 | |
| Nair, Dandotiya et al. (2019) | D | B | C | | A | | A | | C | | A | | 5 | |
| Bobhate and Shrivastava (2011) | C | B | C | | A | | B | | C | | A | | 5 | |
| Patle and Kubde (2014) | C | B | C | | A | | A | | C | | A | | 5 | |
| Rani (2014) | B | A | C | | A | | A | | C | | A | | 7 | |
| Zaidi, Sivakami et al. (2015) | C | B | C | | A | | A | | C | | B | | 4 | |
| Patil (2013) | C | B | C | | B | | A | | C | | B | | 3 | |
| Salve, Dase et al. (2012) | C | B | C | | A | | A | | C | | A | | 5 | |
| Hema Priya, Nandi et al. (2017) | A | A | A | | A | | A | | C | | A | | 8 | |
| Sharma, Mehra et al. (2017) | A | A | A | | A | | A | | C | | A | | 8 | |
| Juyal, Kandpal et al. (2012) | A | A | C | | A | | B | | C | | A | | 7 | |
| Dixit, Raghunath et al. (2016) | A | A | C | | A | | A | | C | | A | | 7 | |
| Deshpande, patil et al. (2018) | C | A | C | | B | | A | | C | | B | | 4 | |
| Mohite and Mohite (2016) | A | A | C | | A | | B | | C | | A | | 7 | |
| Prajapati and Patel (2015) | B | A | C | | A | | A | | C | | A | | 7 | |
| Udayar, Kruthika et al. (2016) | B | B | C | | B | | A | | C | | A | | 5 | |
| Nayak, Toppo et al. (2016) | C | B | C | | B | | B | | C | | B | | 3 | |
| Kansal, Singh et al. (2016) | A | A | A | | A | | A | | B | | A | | 9 | |
| Mahajan and Kaushal (2017) | A | A | B | | B | | A | | C | | A | | 6 | |
| Dixit (2018) | C | B | C | | A | | A | | C | | A | | 5 | |
| Nath and John (2019) | A | A | C | | A | | A | | C | | A | | 7 | |
| Sandhya Rani Javalkar (2017) | A | A | C | | A | | A | | C | | B | | 6 | |
| Bhattacharya, Sen et al. (2015) | B | B | C | | A | | A | | C | | A | | 6 | |
| Prajapati, Shah et al. (2015) | C | A | C | | B | | A | | C | | B | | 4 | |
| Devi, Sivagurunathan et al. (2016) | C | B | B | | A | | B | | C | | B | | 4 | |
| Vijaysree, Kusneniwar et al. (2016) | A | B | A | | A | | A | | C | | A | | 7 | |
| Senapathi and Kumar (2018) | B | B | C | | A | | B | | C | | A | | 6 | |
| Bachloo, Kumar et al. (2016) | A | B | C | | A | | A | | C | | A | | 6 | |
| Rokade and Kumavat (2016) | A | A | C | | A | | A | | C | | A | | 7 | |
| Gupta, Tiwari et al. (2015) | C | B | C | | A | | A | | C | | A | | 5 | |
| Mitra, Mahajan et al. (2015) | A | A | C | | A | | A | | C | | A | | 7 | |
| Agarwal, Fancy et al. (2017) | B | B | C | | A | | A | | C | | B | | 5 | |
| Sridhar and Gauthami (2017) | A | A | C | | A | | A | | C | | A | | 7 | |
| Seenivasan, Priya et al. (2016) | B | A | C | | B | | A | | C | | A | | 6 | |
| Kailasraj, Basavaraju et al. (2020) | C | B | C | | B | | A | | C | | B | | 3 | |
| Santra (2017) | A | A | C | | A | | A | | C | | A | | 7 | |
| Sharma, Choudhary et al. (2016) | D | B | C | | A | | A | | C | | B | | 4 | |
| Srinivasan, Agarwal et al. (2019) | D | A | C | | A | | A | | C | | A | | 6 | |
| **QUALITY ASSESSMENT OF INTERVENTION BASED STUDIES** | | | | | | | | | | | | | | |
|  | **SELECTION** | | | | | **COMPARABILITY** | | **OUTCOME** | | | | | |  |
| **Author, year** | **Representativeness of the exposed cohort**  A) Truly representative of the average in the community *  B) Somewhat representative of the average in the community. *  C) Selected group  D) No description | **Selection of the non exposed**  **cohort**  A) Drawn from the  same community as  the exposed cohort  *  B) Drawn from a  different source  C) No description of the derivation of the non exposed  cohort | | **Ascertainment**  **of exposure**  A) Secure record  (eg clinical  records) *  B) Structured  interview *  C) Written self  report  D) No  description | **Demonstration that outcome of interest was not present at**  **start of study (for side effects)**  A) Yes *  B) No | **Comparability of**  **cohorts on the basis of the design or analysis**  A)Most important  factors of adjustment*  B) Any additional factors * | | **Assessment**  **of outcome**  A)Independent blind assessment *  B) Record  linkage *  C) Self report  D) No  description | | **Was follow- up long enough for outcomes to occur**  A) Yes*  B) No | | **Adequacy** **of**  **follow up of cohorts**  A) Complete  follow up - all  subjects  accounted for *  B) Subjects lost  to follow up  unlikely to  introduce bias *  C) Subjects lost  to follow up >  5% and  description  provided of  those lost  d) No statement | |  |
| Kaur, Vadivukkarassi et al. (2014) | A | A | | B | B | A | | B | | A | | D | | 7 |
| Dwivedi, Sharma et al. (2020) | B | A | | B | A | B | | B | | A | | D | | 7 |
| Premila, Ganesh et al. (2015) | C | A | | B | A | A | | B | | A | | A | | 7 |
| Sahoo, Das et al. (2021) | C | A | | B | A | A | | B | | B | | A | | 6 |
| Singh, Gupta et al. (2020) | C | A | | B | A | A | | C | | A | | A | | 6 |
| Sharma, negi et al. (2015) | B | A | | C | A | A | | B | | A | | A | | 7 |
| Pal, Ahmad et al. (2017) | C | A | | A | A | A | | C | | A | | A | | 6 |

**References**

Agarwal, V., et al. (2017). "Menstrual hygiene: Knowledge and practice among adolescent girls of rural Sabarkantha district." National Journal of Community Medicine**13**(18): 7.0.

Akram, W. and T. Jain (2019) "Knowledge, Attitude, and Practice of menstrual hygiene among adolescent school going girls in Thirumazhisa, Tamil Nadu." Paripex Indian Journal of Research **100**: 0.

Bachloo, T., et al. (2016). "A study on perception and practice of menstruation among school going adolescent girls in district Ambala Haryana, India." Int J Community Med Public Health 3(4): 931-937.

Bathija, G., et al. (2013). "Study on usage of woman hygiene kit among menstruating age group in field practice area of Kims, Hubli." International Journal of Biomedical Research 4(2): 94-98.

Bhattacharyya, M., et al. (2015). "A study of menstrual hygiene among adolescent school girls in a slum area of Kolkata." National Journal of Community Medicine 6(3): 345-348.

Bobhate, P. S. and S. R. Shrivastava (2011). "A cross sectional study of knowledge and practices about reproductive health among female adolescents in an urban slum of Mumbai." Journal of Family and Reproductive Health 12 (5) : 119.

Choudhary, N. and M. K. Gupta (2019). "A comparative study of perception and practices regarding menstrual hygiene among adolescent girls in urban and rural areas of Jodhpur district, Rajasthan." Journal of family medicine and primary care 8(3): 875.

Desai, N., et al. (2018). "Prevalence of polycystic ovary syndrome and its associated risk factors among adolescent and young girls in ahmedabad region." Indian Journal of Pharmacy Practice 11(3): 119.

Deshpande, T. N., et al. (2018). "Menstrual hygiene among adolescent girls–A study from urban slum area." Journal of family medicine and primary care 7(6): 1439.

Devi, R., et al. (2016). "Awareness about menstrual hygiene among adolescent girls in rural area of Kancheepuram District-Tamil Nadu." Int. J. Pharm. Biol. Sci 7: 267-269.

Dixit, A. M. (2018). "A cross-sectional study on menstrual hygiene practices among school going adolescent girls (10-19 years) of Government Girls Inter College, Saifai, Etawah." International Journal of Community Medicine and Public Health 5(10): 4560.

Dixit, S., et al. (2016). "Awareness about menstruation and menstrual hygiene practices among adolescent girls in central India." Natl J Community Med 7(6): 468-473.

Dube, S. and K. Sharma (2012). "Knowledge, attitude and practice regarding reproductive health among urban and rural girls: A comparative study." Studies on Ethno-Medicine 6(2): 85-94.

Dwivedi, R., et al. (2020). "Effect of peer educator-PRAGATI (Peer Action for Group Awareness through Intervention) on knowledge, attitude, and practice of menstrual hygiene in adolescent school girls." Journal of family medicine and primary care 9(7): 3593.

Gopalan, Sarkar., et al. (2020). " Menstrual health knowledge, practices and needs of adolescent school girls". Indian Journal of Maternal and Child Health 15 (1).

Gosavi, S. V., et al. (2015). "Awareness and practices about menstrual hygiene and its impact among migrant adolescent girls of Dera: a community based cross-sectional study from Nashik (Maharashtra)." Int J Sci Rep 1: 123-126.

Gupta, M., et al. (2015). "Awareness and practices regarding menstrual hygiene among women of reproductive age group attending a tertiary care hospital of Indore, India." National Journal of Community Medicine 6(2): 274-277.

Hema Priya, S., et al. (2017). "A study of menstrual hygiene and related personal hygiene practices among adolescent girls in rural Puducherry." Int J Community Med Public Health 4(7): 2348-2355.

Joshi, T., et al. (2015). "Primary dysmenorrhea and its effect on quality of life in young girls." Int J Med Sci Public Health 4(3): 381-385.

Juyal, R., et al. (2014). "Menstrual hygiene and reproductive morbidity in adolescent girls in Dehradun, India." Bangladesh Journal of Medical Science 13(2): 170-174.

Juyal, R., et al. (2012). "Practices of menstrual hygiene among adolescent girls in a district of Uttarakhand." Indian journal of community health 24(2): 124-128.

Kailasraj, K. H., et al. (2020). "A study of knowledge and practice of menstrual hygiene among adolescent school girls in rural and urban field practice area of RajaRajeswari Medical College and Hospital, Bangalore, India." International Journal of Community Medicine and Public Health 7(2):665.

Kansal, S., et al. (2016). "Menstrual hygiene practices in context of schooling: A community study among rural adolescent girls in Varanasi." Indian journal of community medicine: official publication of Indian Association of Preventive & Social Medicine 41(1): 39.

Kaur, K., et al. (2014). "A Pre-Experimental Study to Assess the Effectiveness of Planned Teaching Programme on Knowledge Regarding Menstrual Hygiene Among Adolescent Girls in Selected Schools of Mohali, Punjab." International Journal of Nursing Education and Research 2(4): 362-368.

Kavitha, V. (2012). "Reproductive Health and Hygiene among Adolescents." Language in India **12**(2).

Kulkarni, M. V. and P. Durge (2011). "Reproductive Health Morbidities among Adolescent Girls: Breaking the Silence!" Studies on Ethno-Medicine 5(3): 165-168.

Kumar, D., et al. (2013). "Menstrual pattern among unmarried women from Northern India." Journal of clinical and diagnostic research: JCDR 7(9): 1926.

Kumbhar, S. K., et al. (2011). "Prevalence of dysmenorrhea among adolescent girls (14-19 yrs) of Kadapa district and its impact on quality of life: A cross sectional study." National Journal of Community Medicine 2(2): 265-268.

Kural, M., et al. (2015). "Menstrual characteristics and prevalence of dysmenorrhea in college going girls." Journal of family medicine and primary care 4(3): 426.

Lakkawar, N. J., et al. (2014). "A study of menstrual disorders in medical students and its correlation with biological variables." Sch. J. App. Med. Sci 2(6E): 3165-3175.

Mahajan, A. and K. Kaushal (2017). "A descriptive study to assess the knowledge and practice regarding menstrual hygiene among adolescent girls of Government School of Shimla, Himachal Pradesh." CHRISMED Journal of Health and Research 4(2): 99.

Makapur, V., et al. (2019). " Menstrual hygiene in school going girls : A survey study." International Journal of Research in AYUSH and Allied Systems 7(6) : 2305.

Mathiyalagen, P., et al. (2017). "A descriptive cross-sectional study on menstrual hygiene and perceived reproductive morbidity among adolescent girls in a union territory, India." Journal of family medicine and primary care 6(2): 360.

Misra, P., et al. (2013). "A community-based study of menstrual hygiene practices and willingness to pay for sanitary napkins among women of a rural community in northern India." Natl Med J India 26(6): 335-337.

Mitra, A., et al. (2015). "Awareness and practices on menstrual hygiene amongst adolescent girls in Rajkot district of Gujarat." Healthline Journal 375: 93.75.

Mohite, R. V. and V. R. Mohite (2013). "Correlates of the menstrual problems among rural college students of Satara district." Al Ameen Journal of Medical Science 6(3): 213-218.

Mohite, R. V. and V. R. Mohite (2016). "Menstrual hygiene practices among slum adolescent girls." Int J Community Med Public Health 3(7): 1729-1734.

Nair, A., et al. (2019). "A study to assess the knowledge regarding practices of menstrual hygiene and reproductive tract infections among school going adolescent girls." International Journal of Medical Science and Public Health : 3.

Nath, K. R. and J. John (2019). "Menstrual Hygiene Practices among adolescent girls in a rural area of Kanyakumari District of Tamilnadu." Indian Journal of Youth and Adolescent Health (E-ISSN: 2349-2880) 6(1): 8-14.

Nayak, S., et al. (2016). "A study on practices regarding menstrual hygiene among adolescent girls of urban areas of Jabalpur District." International Journal of Medical Science and Public Health 5(11): 1-3.

Negi, P., et al. (2018). "Menstrual abnormalities and their association with lifestyle pattern in adolescent girls of Garhwal, India." Journal of family medicine and primary care 7(4): 804.

Pal, J., et al. (2017). "Impact of health education regarding menstrual hygiene on genitourinary tract morbidities: an intervention study among adolescent girl students in an urban slum." International Journal of Research in Medical Sciences 5(11): 4937-4941.

Pandit, S. B. (2014). "Common Menstrual Problems among Adolescent students." Sinhgad e Journal of nursing 4.

Paria, B., et al. (2014). "A comparative study on menstrual hygiene among urban and rural adolescent girls of West Bengal." Journal of family medicine and primary care 3(4): 413.

Patil, R. (2013). "Menstrual hygiene and practices of rural adolescent girls of Raichur." Int J Biol Med Res 4(2): 3014-3017.

Patle, R. and S. Kubde (2014). "Comparative study on menstrual hygiene in rural and urban adolescent girls." International Journal of Medical Sciences and Public Health 3 (2) : 129.

Prajapati, D. J., et al. (2015). "Menstrual hygiene: knowledge and practice among adolescent girls of rural Kheda district." National Journal of Community Medicine**13**(10): 50.

Prajapati, J. and R. Patel (2015). "Menstrual hygiene among adolescent girls: A cross sectional study in urban community of Gandhinagar." The Journal of Medical Research 1(4): 122-125.

Premila, E., et al. (2015). "Impact of planned health education programme on knowledge and practice regarding menstrual hygiene among adolescent girls studying in selected high school in Puducherry." Asia Pacific Journal of Resesrch 1(5).

Priyadarshini, S. and S. Shetty (2014). "Dysmenorrhoea among adolescent girls-characteristics and symptoms experienced during menstruation." Journal of Health and Allied Sciences NU 4(03): 045-052.

Rana, B., et al. (2015). "An assessment of menstrual hygiene practices amongst adolescent females at Kheda district of Gujarat state, India." Healthline J 6(1): 23-29.

Randhawa, J. K., et al. (2016). "Effect of dietary habits and socio-economic status on menstrual disorders among young females." Am J Biosci 4: 19-22.

Rani, P. (2014). "Knowledge and practices of menstrual hygiene among married adolescents and young women in chittoor district of Andra Pradesh: India." J Nurs Health Sci 3(2): 06-15.

Ray, S., et al. (2011). "Menstrual characteristics: a study of the adolescents of rural and urban West Bengal, India." Annals of human biology 37(5): 668-681.

Rokade, H. G. and A. P. Kumavat (2016). "Study of menstrual pattern and menstrual hygiene practices among adolescent girls." Natl. J. Community Med 7: 398-403.

Rupa Vani, K., et al. (2013). "Menstrual Abnormalities in School Going Girls–Are They Related to Dietary and Exercise Pattern?" Journal of clinical and diagnostic research: JCDR 7(11): 2537.

Sahoo, Das ., et al. (2021). " Effectiveness of planned teaching program knowledge regarding menstrual hygiene, use of sanitary pads and its disposal management among the adolescent girls in a selected school". European Journal of Pharmaceutical and Medical Research 08 (02) :448-452.

Salve, S., et al. (2012). "Assessment of knowledge and practices about menstrual hygiene amongst rural and urban adolescent girls–A comparative study." Int J Recent Trends Sci Technol 3(3): 1-3.

Sandhya Rani Javalkar, A. K. (2017). "Menstrual hygiene practices among adolescent schoolgirls of rural Mangalore, Karnataka." International Journal of Medical Science and Public Health 6(7).

Santra, S. (2017). "Assessment of knowledge regarding menstruation and practices related to maintenance of menstrual hygiene among the women of reproductive age group in a slum of Kolkata, West Bengal, India." Int J Community Med Public Health 4(3): 708-712.

Sarkar, A. P., et al. (2016). "Premenstrual syndrome among adolescent girl students in a rural school of West Bengal, India." Int J Med Sci Public Health 5(03): 5-8.

Seenivasan, P., et al. (2016). "Knowledge, attitude and practices related to menstruation among adolescent girls in Chennai." J. Clin. Sci. Res 5: 164-170.

Senapathi, P. and H. Kumar (2018). "A comparative study of menstrual hygiene management among rural and urban adolescent girls in Mangaluru, Karnataka." International Journal of Community Medicine and Public Health 5(6): 2548.

Shah, M., et al. (2013). "A study of prevalence of primary dysmenorrhea in young students-A cross-sectional study." Healthline 4(2): 30-34.

Shanbhag, D., et al. (2012). "Perceptions regarding menstruation and practices during menstrual cycles among high school going adolescent girls in resource limited settings around Bangalore city, Karnataka, India." International Journal of Collaborative Research on Internal Medicine & Public Health 4(7): 1353.

Sharma, R., et al. (2015). "Menstrual hygiene among adolescent girls." Indian journal of community health 27(3): 376-380.

Sharma, S., et al. (2016). "Knowledge and practices about menstrual hygiene among school adolescent girls in Agroha village of Haryana." Journal of Evolution of medical and Dental Sciences 5(9): 389-393.

Sharma, S., et al. (2017). "Menstrual hygiene practices among adolescent girls in a resettlement colony of Delhi: a cross-sectional study." Int J Reprod Contracept Obstet Gynecol 6(5): 1945-1951.

Singh, A., et al. (2013). "Knowledge, attitude and practices about menstruation among adolescent female in Uttarakhand." Panacea J Med Sci **3**: 19-22.

Singh, A., et al. (2020). "A cross-sectional study to investigate the impact of focused group discussion on menstrual hygiene among rural school girls of Southern Haryana, India." Journal of Education and Health Promotion 9.

Singh, M., et al. (2019). "Menstrual patterns and problems in association with body mass index among adolescent school girls." Journal of family medicine and primary care 8(9): 2855.

Singh, A., et al. (2013). "Knowledge, attitude and practices about menstruation among adolescent female in Uttarakhand." Panacea J Med Sci **3**: 19-22.

Singh, Z. and S. Datta (2014). "Perception and practices regarding menstruation among adolescent school girls in Pondicherry." The Health Agenda **2**(4).

Sridhar, D. and N. Gauthami (2017). "Menstrual health status and cultural practices of tribal adolescent girls." Int J Commun Med Publ Health 4(11): 4120-4124.

Srinivas, P. (2016). "Perception, knowledge and practices regarding menstruation among School going girls in Karaikal." IOSR J Dent Med Sci 15(1): 27-34.

Srinivasan, D., et al. (2019). "Awareness, perceptions and practices regarding menstruation and menstrual hygiene among students of a college in Bengaluru Urban district, South India: a cross sectional study." International Journal of Community Medicine and Public Health 6(3): 1126-1132.

Thakre, S. B., et al. (2011). "Menstrual hygiene: knowledge and practice among adolescent school girls of Saoner, Nagpur district." J Clin Diagn Res 5(5): 1027-1033.

Tripathi, Aggarwal., et al (2021)." Prevalance and associated risk factors of polycystic ovarian disease in professional college going girls of agra city: a cross- sectional study". International Journal of Community Medicine and Public Health 8(4) : 1917.

Udayar, S. E., et al. (2016). "Menstrual hygiene practices among adolescent girls residing in tribal and social welfare hostel in Andhra Pradesh: a community based study." Natl J Commun Med 7(8): 681-685.

Varghese, M. M., et al. (2015). "Religious restrictions and cultural taboos related to menstruation in adolescent girls: a school-based cross-sectional observational study." Indian Journal of Child Health 2(4): 161-164.

Verma, P., et al. (2013). "Knowledge and practices about menstrual hygiene among higher secondary school girls." Indian journal of community health 25(3): 265-271.

Vijaysree, L., et al. (2016). "A study on menstrual practices and hygiene among adolescent girls in a government high school." Indian J Med Res Pharm Sci 3: 55-60.

Yadav, A. and D. L. Masand (2018). "F Study of menstrual disorder in adolescent girls at tertiary care centre in rural area." Int J Reprod Contracept Obstet Gynecol 7: 1979-1983.

Zaidi, S. H. N., et al. (2015). "Menstrual hygiene and sanitation practices among adolescent school going girls: a study from a South Indian town." International Journal of Community Medicine and Public Health 2 (2) : 189.
